# Long-term concentration of tropical forest nutrient hotspots is generated by a central-place apex predator

**DOI:** 10.1038/s41598-023-31258-8

**Published:** 2023-03-17

**Authors:** Everton B. P. de Miranda, Carlos A. Peres, Luiz Gustavo Rodrigues Oliveira-Santos, Colleen T. Downs

**Affiliations:** 1 The Peregrine Fund, 5668 West Flying Hawk Lane, Boise, ID 83709 USA; 2 Centre for Functional Biodiversity, School of Life Sciences, University of KwaZulu-Natal, P/Bag X01, Pietermaritzburg, 3209 South Africa; 3 School of Environmental Sciences, University of East Anglia, Norwich, NR47TJ UK; 4 Instituto Juruá, Manaus, Brazil; 5 Programa de Pós-Graduação em Ecologia e Conservação, Universidade Federal de Mato Grosso do Sul, Av. Costa e Silva, Campo Grande, MS 79070-900 Brazil

**Keywords:** Ecology, Biogeochemistry, Ecology

## Abstract

Apex predators typically affect the distribution of key soil and vegetation nutrients through the heterogeneous deposition of prey carcasses and excreta, leading to a nutrient concentration in a hotspot. The exact role of central-place foragers, such as tropical raptors, in nutrient deposition and cycling, is not yet known. We investigated whether harpy eagles (*Harpia harpyja*) in Amazonian Forests—a typically low soil fertility ecosystem—affect soil nutrient profiles and the phytochemistry around their nest-trees through cumulative deposition of prey carcasses and excreta. Nest-trees occurred at densities of 1.5–5.0/100 km^2^, and each nest received ~ 102.3 kg of undressed carcasses each year. Effects of nests were surprisingly negative over local soil nutrient profiles, with soils underneath nest-trees showing reductions in nutrients compared with controls. Conversely, canopy tree leaves around nests showed significant 99%, 154% and 50% increases in nitrogen, phosphorus and potassium, respectively. Harpy eagles have experienced a 41% decline in their range, and many raptor species are becoming locally extirpated. These are general examples of disruption in biogeochemical cycles and nutrient heterogeneity caused by population declines in a central-place apex predator. This form of carrion deposition is by no means an exception since several large raptors have similar habits.

## Introduction

In his seminal work (Animal Ecology, 1927^1^), Charles Elton states: “It is usual to speak of an animal as living in a certain physical and chemical environment, but it should always be remembered that strictly speaking we cannot say exactly where the animal ends and the environment begins”. Elton meant that animals can only be interpreted if their ecological interactions are considered. This was followed by a profusion of twentieth-century ecological research built around this idea. Elton then continued: “unless it is dead, in which case it has ceased to be a proper animal at all”. In the twenty first century, ecologists have explored in detail the notion that even after death, animals continue to influence their physical and chemical environments, sometimes at unexpected spatial scales^2^. Nothing exerts a stronger connection between life and death, the two conditions discussed by Elton, than an apex predator^3^. In particular, the rapacious nature of apex predators renders them inextricably intertwined with their chemical and physical environments.

Animals can influence biogeochemical cycles via two main processes: concentration of nutrients into hotspots^4^ and diffusion of nutrients against natural gradients^5^. Since big and fierce animals are rare^6^, concentration in hotspots is the main pathway through which they influence biogeochemical cycles. Nutrient subsidies can be defined as a donor-controlled resource from one habitat to a recipient (such as a plant) in another habitat. This increases the productivity (as population growth or larger body size) of the recipient, potentially altering the consumer–resource relationship in the recipient ecosystem^7^. For apex predators, those resources are usually prey carcasses or prey-derived detritus (scats or excreta) that are locally concentrated because of landscape traits^4,8^.

Amazonian forest soils are susceptible to changes in the distribution of key nutrients because they are usually nutrient-poor^9^, as most nutrients are generally concentrated in the aboveground forest biomass. Modest changes in soil nutrient profiles can profoundly affect biodiversity, as in mineral licks visited by geophagous vertebrates in search of sodium^10^. However, good apex predator candidates for the role of nutrient concentrators in the megadiverse Amazonian ecosystem are hard to predict. Apex predators include black caiman (*Melanosuchus niger*), green anaconda (*Eunectes murinus*), puma (*Puma concolor*), jaguar (*Panthera onca*) and harpy eagle (*Harpia harpyja*)^11^. Therefore, single-species effects over any phenomena should be rare or non-existent as predicted by ecological theory applied to tropical ecosystems^12^.

**Figure 1:**
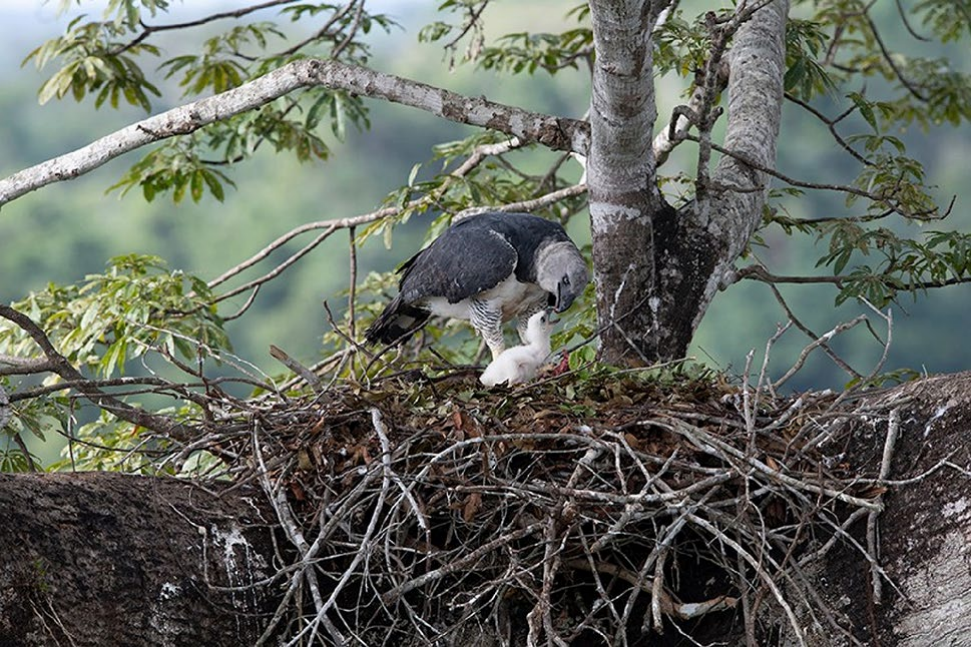
Harpy eagle female protecting an eaglet at her nest in an emergent kapok tree (*Ceiba pentandra,* Bombacaceae). Photo: Barbara Fleming.

Harpy eagles (Fig. 1) are particularly interesting in their nutrient concentration role. They typically nest in the same giant emergent tree for decades^13^. The harpy eagle's breeding cycle is the longest of all birds, during which they bring prey to their eaglets for 30–36 months^13^. They feed extensively on medium-sized canopy vertebrates^14^. Bird excreta is often rich in limiting nutrients^15^, and harpy eagle excreta often taints the nest tree's surrounding canopy foliage and branches. Harpy eagles are, therefore, central place foragers, as they maximise foraging rates while travelling through a discrete resource concentration while maintaining the key distinction of a forager travelling from a home base (the nest tree) to a distant foraging location^16,17^. These traits render them an ideal model to test the nutrient concentration hypothesis in Amazonian forests. Although harpy eagles are Earth’s largest eagles, they are surpassed by other apex predators that suit the role of affecting nutrient distribution in the landscape—such as wolves and bears—by one or two orders of magnitude in body mass^18^. Contrary to those predators, harpy eagles do not rely on landscape traits to increase prey kill rate and are obligatory central-place foragers once they reach breeding age. This raises the question: can harpy eagles influence soil chemistry and, therefore, nutrient cycling in their ecosystem?

**Figure 2:**
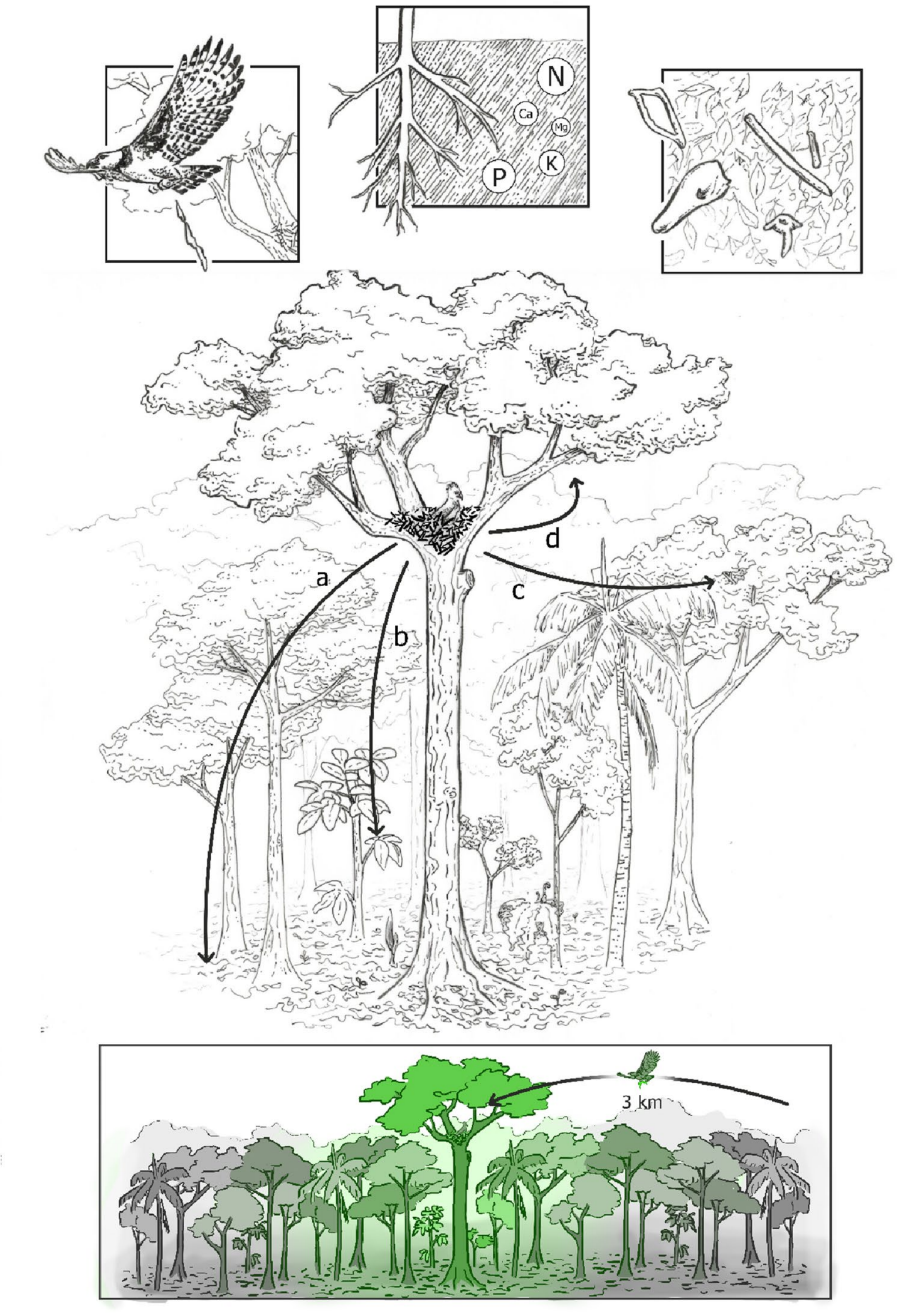
Schematic representation of the nutrient concentration role of nesting harpy eagles through central-place prey carcass and excreta deposition on different vegetation strata, including soil (**a**), the understorey foliage (**b**), canopy (**c**) and the nest tree itself. Lower panel represents the landscape-scale dynamic of nutrient concentration from commuting distances of up to 3 km from the nest. Magnitude of nutrient concentration is indicated by increasingly green colours. Illustration: Paula Viana.

Our objectives were to test the hypothesis that harpy eagles serve as accumulation agents of nutrients by concentrating decaying remains of prey items at nest sites, thereby biomagnifying soil and foliage nutrients in the undergrowth, canopy vegetation and nest-tree (Fig. 2). We predicted that harpy eagles would increase the nutrient profiles of both soils and foliage. In providing these ecosystem-level insights, we attempt to show how local extinctions of this apex predator may result in disruptions in biogeochemical cycles that modulate soil and vegetation nutrient heterogeneity across the Amazon Basin.

## Results

We collected soil and vegetation samples from 20 harpy eagle nests; 10 active, 10 inactive, plus one nest that we sampled while both active and inactive. Those 21 samples were paired with 47 conspecific control trees at which we collected comparable soil and vegetation samples. Nest tree species included 16 Brazil nut trees (*Bertholletia excelsa* Lecythidaceae), one self-standing *Ficus* spp. (Moraceae), one *Astronium lecointei* (Anacardiaceae), one *Cariniana* spp. (Lecythidaceae) and one *Apuleia leiocarpa* (Fabaceae). Although we selected the largest available emergent individuals for non-nest trees, tree diameter at breast height (dbh) was significantly larger in nest trees (mean ± SD, 148.3 ± 25.5 cm) than in control trees (134 ± 25 cm; β = 0.62, t = 5.94, *p* < 0.01).

### Carcass input

Using camera-traps to monitor 10 harpy eagle nests (or 20 adult eagles), we recorded 212 prey items amounting to an estimated 411 kg of prey delivered per nest per nesting cycle (Table 1). Although adults continue to deliver prey at low rates to their nests after 24 months, eaglets usually consume these elsewhere, in addition to hunting independently. We, therefore, labelled nesting cycles older than 24 months as “nutrient-inactive” and excluded these data from our carcass biomass input estimates. This resulted in a total of 307 kg of prey delivered per nesting cycle (36 months), or approximately 102.3 kg per year.

**Table 1:** Prey delivery rates of harpy eagles per nesting phase.

Nesting phase (months)	Day/prey	Range	Prey mass (kg)	kg/day	Prey deliveries (n)	Adult eagles (n)
Unfledged (0–6)	1.8 ± 0.92	0–3	1.11 ± 1.06	0.62	11	6
Early fledged (7–12)	2.5 ± 1.71	0–8	1.33 ± 1.18	0.53	87	6
Late fledged (13–24)	4.17 ± 3.02	0–15	1.16 ± 0.86	0.28	36	4
Dispersing (25–36)	5.12 ± 4.90	0–21	1.49 ± 0.89	0.29	78	10
Whole cycle				411.52 kg		
Last year excluded				306.95 kg		

The four phases represent different stage on which eaglets (and adult females, in case of early-unfledged eaglets) receive prey on the nest. Since eagles > 24 months frequently eat prey out of the nest, and hunt independently, those were removed from input calculation. Prey delivery rates (day/prey) and biomass delivery rates (kg/day) are shown in mean ± SD. Range represents the number of days between prey deliveries, with 0 days representing two deliveries on a single day.

Using the maximum packed nest density, we estimated 1.97–4.84 nests/100 km^2^ throughout our study area (Supplementary Information, Table S1, Fig. S1). The polygon method produced densities of 1.55–3.30 nests/100km^2^. Consequently, as a central-place hunter, harpy eagles concentrated 102.3 kg/year of prey captured over 20–64 km^2^ into a single carcass hotspot, over an approximate density of 1.5–5.0 carcass hotspots per 100 km^2^.

### Soil nutrients

**Figure 3:**
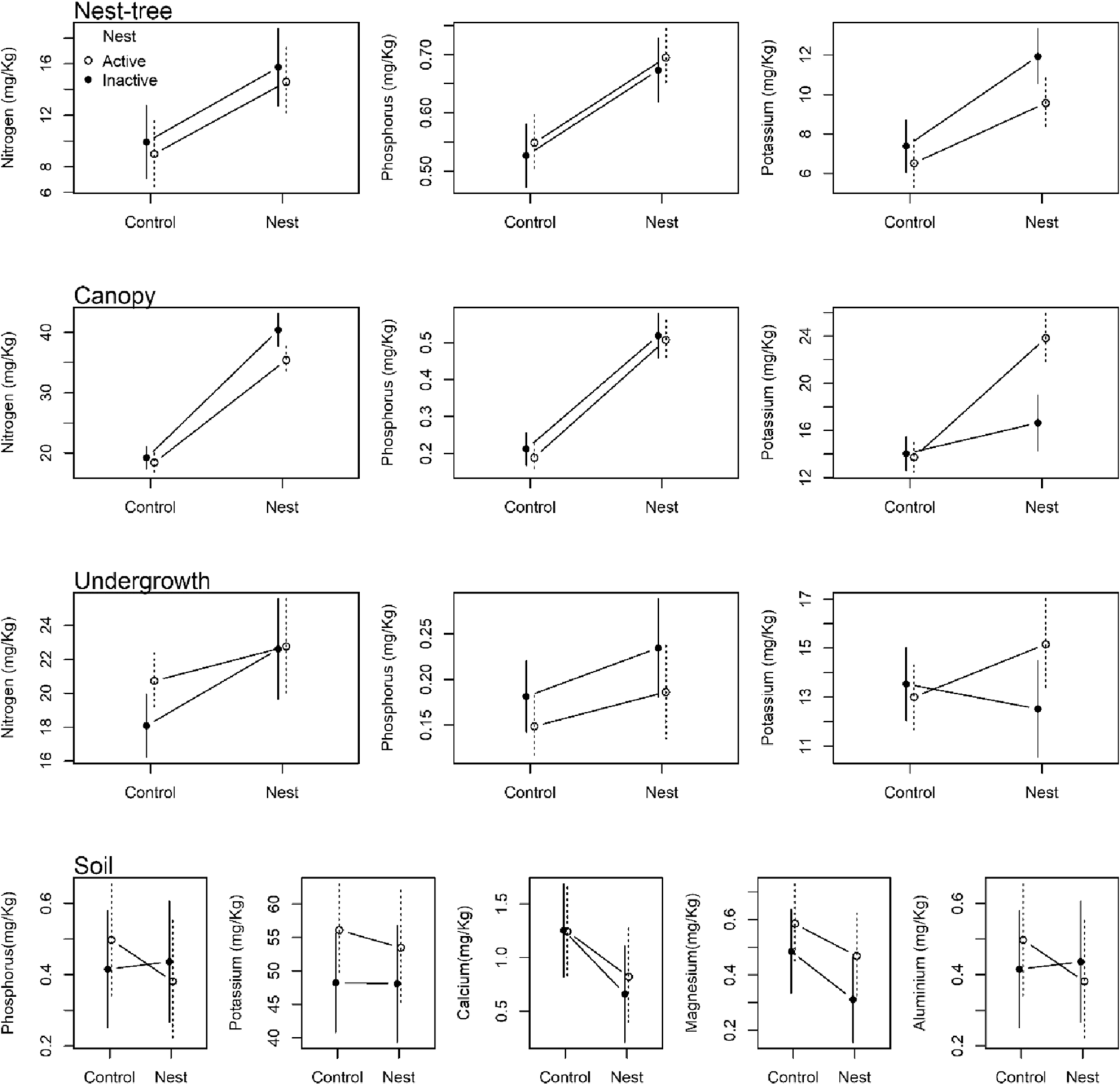
Effects of long-term presence of harpy eagle nests on the nutrient profile in both the soils and across the forest vertical stratification depending on nest activity (were white circles mean active and black circle mean inactive).

Contrary to our expectations (Supplementary Information Table S2: soil stratum; Fig. 3: soil panel), four of the five measured soil nutrients underneath nest trees had a reduced concentration compared to controls: (1) phosphorus (50% reduction; β =  − 0.11, t =  − 2.29, *p* < 0.03); (2) calcium (32% reduction; β =  − 0.42, t =  − 3.07, *p* < 0.01); (3) magnesium (21% reduction; β =  − 0.11, t =  − 2.71, *p* < 0.01); (4) aluminium (50% reduction; β =  − 0.08, t =  − 2.29, *p* < 0.03); (5) while not for potassium (β =  − 2.65, t =  − 0.67, *p* = 0.50). We did not detect any main effect of nest activity for any nutrient type (*p* > 0.05; i.e. nest effects were detected irrespective of activity), nor any interaction between nest presence and nest activity (*p* > 0.05). Positive effects of tree size (i.e. diameter at breast height) were important as a covariate for phosphorus (β = 0.13, t = 1.83, *p* < 0.01), potassium (β = 3.68, t = 2.27, *p* < 0.03) and aluminium (β = 0.06, t = 2.62, *p* < 0.01), but not for the other nutrients (*p* > 0.05).

### Understorey vegetation

We detected an effect of eagle nest presence only for potassium concentration (16% increase; β = 2.15, t = 2.22, *p* < 0.03) in the foliage samples in undergrowth vegetation (Supplementary Information Table S2: undergrowth stratum, Fig. 3: undergrowth panel). Nitrogen (β = 2.03, t = 1.23, *p* = 0.22) and phosphorous (β = 0.03, t = 1.35, *p* = 0.17) concentrations were unaffected by nest presence. We did not find an effect of nest activity on any nutrient concentration (*p* > 0.05), nor the interaction between nest presence and nest activity (*p* > 0.05; Table S2), except for potassium. In the case of potassium concentration, a positive effect of nest presence occurred around active nests but not around inactive nests (interaction term; β =  − 3.17, t =  − 2.29, *p* < 0.03). Tree girth was also unimportant in determining the magnitude of nutrient concentrations (*p* > 0.05).

### Forest canopy around nest-trees

Nest presence had a positive effect on foliage nutrient concentration of canopy trees adjacent to nest-trees for nitrogen (87% increase; β = 16.95, 13.03, *p* < 0.01), phosphorus (142% increase; β = 0.31, t = 12.24, *p* < 0.01), and potassium (79% increase; β = 10.11, t = 8.32, *p* < 0.01; Supplementary Information Table S2: surrounding canopy trees, Fig. 3: canopy panel). However, although we failed to detect a main effect of nest activity per se (*p* > 0.05), nest activity magnified the positive effect of nests on the concentration of nitrogen (interaction term; β = 4.20, t = 1.95, *p* < 0.04) and potassium (interaction term; β = 7.50, t = 4.09, *p* < 0.01), but not phosphorus (interaction term; β =  − 0.01, t =  − 0.31, *p* = 0.75). Nest activity also magnified the positive effect of nests on the canopy foliage for nitrogen and phosphorus by 24% and 74%, respectively, as observed for potassium. Tree size was unrelated to any of the foliar nutrients (*p* < 0.05).

### Nest-trees

Long-term presence of harpy eagles at the nest exerted a strong positive effect on nutrient concentrations of nest-tree foliage for nitrogen (80% increase; β = 5.60, t = 9.73, *p* < 0.01), phosphorus (25% increase; β = 0.14, t = 23.54, *p* < 0.01), and potassium (47% increase; β = 3.04, t = 12.44, *p* < 0.01) (Table S2: nest tree; Fig. 3: nest-tree panel). However, we did not detect a main effect of nest activity (*p* > 0.05) on foliage nutrient concentrations, nor the interaction between nest presence and nest activity (*p* > 0.05), with the exception of potassium. For potassium, nest activity amplified the positive effect of nest presence by 50% (interaction term; β = 1.51, t = 4.13, *p* < 0.01). There were no effects of tree girth on any of the nutrient levels (*p* < 0.05).

## Discussion

Apex predators exert decisive effects on prey distribution, demography and behaviour^11,19^, but whether these extend to the base of food-webs remains uncertain. Here, we have shown that a rare central-place apex carnivore enhances nutrient availability for local plant communities in Amazonian forests. These findings are of prime interest to food web and carrion ecology. Our study highlights a new mechanism through which an apex predator exhibiting long-term site-fidelity can affect soil heterogeneity. This process is driven by increasing key nutrients in vegetation through the cumulative deposition of both carrion and excreta that is neither ephemeral nor constrained by landscape traits. This site-specific trophic connectivity, although now seemingly obvious, has remained hitherto undocumented because the ecology of raptor and plant communities represent opposite extremes of food webs and may appear to be hardly intertwined.

Our estimates of harpy eagle nest densities of 1.5–5.0 nests/100 km^2^ allow us to hypothesize that these effects are important at the landscape scale, generating a heterogeneous nutrient pump that favours the uptake of rare nutrients into the vegetation on otherwise oligotrophic Amazon soils. Subalusky and Post^20^ highlighted that carcass input rates are one of the most frequently missing links in carcass subsidy studies. The direct and sustained nutrient carcass inputs per nest tree shown here—roughly 102.3 kg per year—challenges one of the central notions in carcass ecology: that the effects of animal carcasses are ephemeral^21^. We have shown a mechanism by which carcasses are consistently deposited at the same spot for periods that may extend for several decades. This deposition is conditional on the harpy eagle's reproductive phenology, which fluctuates over time, resulting in different vegetation nutrient profiles between active and inactive nest sites. Nest activity resulted in 24% and 74% increases in nitrogen and phosphorus in canopy foliage around the nest-tree, and an 50% increase in potassium for the nest-tree itself. The “rare and unpredictable” availability of large vertebrate carrion is therefore challenged by this study, as seen in the consistent and frequent nutrient deposition through space and time of an otherwise limited resource.

Carrion is known to result in intense interspecific competition, and this form of interaction has been shown to occur at harpy eagle nests^22^. This mechanism of carrion deposition is widespread in raptors, even if the magnitude of carcass deposition by smaller raptors that consume smaller prey is more modest. On the other hand, several large-bodied raptors are known to exhibit similar philopatric habits, including bald (*Haliaeetus leucocephalus*), crowned (*Stephanoaetus coronatus*), martial (*Polemaetus bellicosus*), golden (*Aquila chrysaetos*), and Philippine (*Pithecophaga jefferyii*) eagles^23–26^. This poses the question of how these species affect nutrient mosaics of soils in their range.

The amount, quality and duration of carcass inputs are influenced by how the input takes place. Prey remains underneath harpy eagle nests are mostly skeletal material, with few or no soft body parts. Bones are the main sources of phosphorus but exhibit the slowest decomposition rate of all carcass parts, taking 170 times longer than muscle to decompose in large mammalian carcasses^27^. Harpy eagles primarily prey on medium-sized vertebrates (which have a lower proportion of phosphorus^28^), and arboreal termites often cover skeletal remains within hours after they fall to the ground. This likely accelerates assimilation rates (EPBM, pers. obs.), and consequently provides relatively little opportunity for the diffusion of phosphorus and other nutrients into the topsoil. On the other hand, harpy eagles regularly provide soluble nutrient-rich excreta, which are easily assimilated by vegetation^29^ through the leaves, making excreta a high-quality resource.

Nevertheless, we found that active harpy eagle nests had a puzzling impact on soil quality, resulting in lower soil nutrient availability. Except for potassium, soil nutrients decreased in soil samples underneath nest-tree crowns (by − 50% for phosphorus, − 32% for calcium, − 21% for magnesium, and − 50% for aluminium). In contrast, other cases of clumped deposition of faecal/excreta material typically boosts soil nutrients. For instance, manganese and potassium concentrations are elevated in soils beneath the latrines of frugivorous spider monkeys^30^ (*Ateles* spp.). Howler monkeys (*Alouatta* spp.)—which are folivore-frugivores—also increase the fertility of soil nutrient profiles, with phosphorus content increasing by 3.8–6.0 times in latrines beneath sleeping sites compared with control sites^31^. Since foliage and fruit pulp are relatively poorer than carcasses in nitrogen and phosphorus^32^, how can these species positively affect soil nutrients while harpy eagles do not?

The strong and consistent response of canopy foliage chemistry to deposition of harpy eagle excreta (i.e. 99%, 154% and 51% increases in nitrogen, phosphorus and potassium, respectively) suggests that the apparent soil nutrient sink we observed is a consequence of canopy trees shortcutting access to excreta nutrients through direct leaf uptake, rather than from the soil per se. Excreta ejected by adults and eaglets usually smears much of the canopy foliage underneath the nest-tree, which is not the case for most detrital resources in tropical forests, such as primate dung, that fall on the ground. By intercepting an important limiting nutrient—phosphorus—before it reaches the ground, canopy trees putatively absorb other nutrients in more significant amounts, including phosphorus. In a classic case of Liebig's Law of the minimum^33^, nest trees likely fill the “barrel gap” through leaf uptake, consequently reducing soil nutrient profiles.

Nest-trees also showed the effects of harpy eagle nesting. While those effects may be moderate compared with the canopy, nest-trees may also remove nutrients from soils underneath their crowns by shortcutting the phosphorus uptake pathway. Some nutrient absorption likely occurs via the bark (the position of harpy eagle nests allows excreta to reach terminal foliage in only 2.1% of nests^34^), but bark nutrient uptake is, at best, poorly understood^35^. This is likely the reason for the weaker and inconsistent increase in the nest-tree foliage nutrient profile compared with the surrounding canopy. Harpy eagle nests are large renewable structures averaging 152 × 99 cm^34^. This large platform of piled dead branches accumulates much of the carcass and skeletal remains while adults continue to add new green branches. This accumulated nutrient source—a metaphorical carrion-enriched compost pile—possibly becomes directly available to the nest-tree through bark absorption, given the runoff of nutrients dissolved in rainwater through > 30 m boles (or an average bark surface of 143.2 m^2^ per nest-tree bole) before reaching the ground. While water absorption through bark has been shown in several trees^36,37^, the degree to which this occurs in tropical emergent trees remains unknown. Nevertheless, this form of nutrient enrichment is the single most likely mechanism for increasing foliar nutrient profiles of nest-trees observed here, thereby enabling a sustained physicochemical mutualism between harpy eagles and their long-term nest-trees. To what degree this may affect the reproductive fitness and longevity of individual emergent “mega-trees”^38^, and thereby ensuring the long-term availability of suitable nest platforms, remains unclear.

Therefore, these findings suggest non-obligatory reciprocal benefits between harpy eagles and their nest-trees. Harpy eagles require particular morphological traits for emergent trees to be selected as suitable nest-trees, particularly exceptionally high crown stature and T-shaped primary branching^34^. Trees with this crown architecture may, in turn, benefit from nesting harpy eagles through the significant, long-term deposition of limiting nutrients. These effects may also feedback to influence the donor ecosystem: direct nutrient augmentation in the canopy may affect the primary tree productivity via increased foliage and fruit production. To what degree active nests increase nest tree demographics, including growth rates, fecundity (seed production) and longevity remain largely open questions. Given the extremely low density of large raptors throughout Neotropical forests^39^, this also creates a patchy mosaic of rare but sustained nutrient hotspots in a forest landscape otherwise dominated by nutrient-poor soils. Since heterogeneity is one of the main spatial correlates of landscape-scale plant and animal diversity, this could represent a mechanism by which the understorey in the vicinities of harpy eagle nests show higher floristic diversity^40^. This illustrates how this trophic cascade generates a wealth of testable hypotheses^41^ for future studies.

The undergrowth vegetation underneath harpy eagle nest trees had higher potassium content even if rooting in nutrient-poor soils. Significant increases of 16% for potassium have also likely resulted from the direct deposition of harpy eagle excreta on leaves, ensuring direct nutrient absorption while circumventing below-ground root uptake. Although we selected understorey foliage samples that lacked clear signs of excreta staining, this may be masked by the detritus runoff during the frequent downpours during the wet season, leaving no evidence of recent animal excreta. Foliar absorption is a well-known phenomenon that occurs in > 85% of all plant species tested^42,43^, and the time-lag required for 50% absorption of phosphorus is estimated at 7–15 days, which is a plausible interval between consecutive rains. Other nutrients have a much shorter absorption time, estimated at 1–24 h for nitrogen and 1–4 days for potassium^42^. Elevated root nutrient uptake is frequently induced by foliar fertilisation in several plant species^44^.

Large hypercarnivores are rare and becoming rarer worldwide^45,46^, so the degree to which local extinctions disrupt ecosystem processes should be explored in further detail. Harpy eagles have succumbed to a 41% decline in their distribution range^47,48^, and have been locally extirpated over vast landscapes of the Brazilian Atlantic Forest and Mesoamerica, which now lack this apex predator and its long-term forest nutrient transport and aggregation, and downstream bottom-up effects on vegetation. The restoration plans dedicated to the species in the Atlantic Forest^47,49^ will provide an opportunity to test how the return of those species affects this phenomenon.

The markedly philopatric nature of large birds of prey suggests that other declining raptor species can perform similar ecological roles. We can only speculate on the magnitude of nutrient hotspots generated by the now-extinct mega-raptors that once hunted many of the World’s large islands. Large raptors that were (pre)historically driven to extinction by humans, such as the Cuban terrestrial owl (*Ornimegalonyx oteroi)* and New Zealand’s Haast eagle (*Hieraaetus moorei*), were much larger than harpy eagles and likely performed similar central-place nutrient inputs into soils and vegetation. This is another example of how historical and ongoing large vertebrate extinctions can sever nutrient transport systems, severely affecting biogeochemical cycles and nutrient redistribution^5^.

In conclusion, we suggest that harpy eagles impact soil and vegetation nutrient heterogeneity by regularly depositing excreta and carrion from their nests over decades. This not only enhances our understanding of the role of natural predation and carrion deposition in sustaining nutrient cycles, but also elucidates the role of large raptors in ecosystem processes. Finally, our findings pose further questions regarding how far the effects triggered by multi-annual site-specific prey delivery by large raptors reverberate over animal and vegetation communities in tropical forests, and the degree to which trophic downgrading results from the increasingly widespread spate of apex-predator extinctions.

## Methods

### Study area

Our study was conducted in the southern Amazon Forest—known in conservation circles as the Arc of Deforestation—in the northern part of the state of Mato Grosso, Brazil (Supplementary information Fig. S2). Koeppen^50^ classifies the regional climate as “tropical wet climate” or Amazonian (tropical monsoon climate). Rainfall averages 2350 mm/yr, and the ambient temperature averages 24.5 °C, combined with a high relative air humidity (80–85%^51^). As in most of the Amazon Basin, soils in our study region are highly acidic^9^. Levels of phosphorus, potassium, calcium, and magnesium are generally low, while aluminium and H + Al—which are toxic to plant life—are generally high. Phosphorus limitation is particularly severe in the region^5^.

### Carcass input

We installed 2–3 camera-traps at each harpy eagle nest site to sample their food delivery rates, thereby enabling estimates of nutrient input into the soil underneath the nests from remains of prey carcasses. To do so, we used nests without signs of food stress resulting from habitat fragmentation. Since prey delivery rates decreased with eaglet development, we defined four categories of prey delivery rates: 0–6 months (unfledged), 7–12 months (recently fledged eaglets), 13–24 months (late-fledged eaglets) and 25–36 months (individuals at the onset of dispersal). We inferred eaglet age from known hatchling dates, or feather colours and nest degradation status when the hatchling date was unknown^52^. We defined nests as unoccupied as those in disuse because the pair was active at an alternative nest (a minority of 16% of harpy eagles have alternative nests;^39^) and those with eaglets above 24 months.

We estimated prey biomass delivered per nest based on the average body size described for each prey species. Subadult prey were attributed two-thirds of the adult body mass. For infant or juvenile prey (such as ungulates that were killed almost exclusively as newborns^53^), we attributed one-fifth of the adult body mass. Sloths received a further reduction of one-third, because of the large amount of foliage representing ~ 30% of the body mass in living sloths^54^ that harpy eagles discard soon after kills. As prey items are usually dismembered when delivered to nests, we applied approximate reductions in body mass as follows: 10% (head or viscera), 20% (per member missing or single-member delivered), 50% (lower or upper body missing), and 90% (tail of Atelinae primates, porcupines) of the total prey body mass^23^.

We also calculated harpy eagle nest density to infer nutrient input rates at the landscape scale. We found harpy eagle nests by actively distributing brochures advertising a financial reward of c. USD100 (BRL500) to anyone who could locate a harpy eagle nest. We found a total of 38 nests over four years. After excluding alternative nests, we calculated active nest density using two methods: maximum packed nest density (MPND), and the polygon method. We selected these methods to maximise comparability with previous studies^39^. The MPND^55^ was calculated as:

$${\text{A}} =\uppi {\text{r}}^{2} *1.158$$

where A is defined as half the distance to the nearest neighbouring nest in a cluster of nests. This distance is then considered as the radius for a circular breeding territory centred at the nest. The 1.158 is a constant designed to fill the interstitial space between breeding territory circumferences. The polygon method uses half the average distance to the nearest neighbouring nest to establish a polygon around all nests within a cluster, from which we estimated the total area. We calculated the two density estimates for our study area based on two known clusters of nests (with five and six nests each). We concentrated the most intensive sampling effort on those clusters over the last four years, thereby deriving a high nest detection rate. We then divided the values that resulted from both estimates by 100 km^2^ of available forest habitat to derive a nest density estimate.

### Soil sampling

We collected five soil samples underneath each harpy eagle nest-tree at 5–15 cm depth using a mechanical auger, at 1–10 m distance from the nest tree, where excreta and prey bones typically fall. We paired each nest-tree with one to three same-species, emergent-sized control trees. Local peculiarities made only one or two control trees of the same species available in some locations. This phylogenetic control was done to ensure that any nutrient effects were related to harpy eagle nesting activity, rather than tree species identity. We added tree circumference at breast height to the models to warrant that the effects were not from the mega-trees since they can alter soil composition by deposition of leaves, bark, and branches. We excluded any emergent trees frequently used as perches by adults and fledged eaglets as control sites.

We quantified soil aluminium, potassium, calcium and magnesium using the 1 M KCl extraction method^56^. Concentrations of these nutrients were determined by atomic absorption spectrophotometry. Phosphorus was extracted using the Mehlich 1 method^57^, and phosphorus concentrations were determined by spectrophotometry at 725 nm^56^.

### Vegetation sampling

At each nest-tree and control tree site, we collected undergrowth vegetation samples randomly at 1–10 m from the focal tree bole. We selected foliage from mature branches of three healthy stems from different individuals of up to 1 m in height that were free from direct signs of harpy eagle excreta. We did not select foliage from any particular plant species. Samples were stored in paper envelopes and dehydrated naturally. We also collected foliage samples from three branches of 25–30 m canopy-height trees at 5–15 m from the nest, as well as foliage from three branches from nest-trees, using a rope chainsaw.

We rinsed vegetation samples in distilled water to remove detrital material and dehydrated them in an oven, and then ground them until homogenised. We analysed the nitrogen, phosphorus and potassium content of each sample using methods described by Embrapa^56^. Nitrogen was extracted using sulphuric acid (total Kjeldahl N). Nitricperchloric extract was used for the other elements: phosphorus (colorimetry) and potassium, which were subsequently determined by spectrophotometry at 725 nm^56^.

### Statistical analyses

We used Generalised Linear Mixed Models to test the effect of nest presence and nest occupancy (0 or 1), as well as their interaction, on nutrient profiles in the soil and leaf samples across the vertical stratification of foliage. We ran a model for each nutrient in each stratum (five nutrients for soil and three nutrients for each forest stratum: foliage in the undergrowth, canopy trees around the nest-tree, and the nest-tree). Because we strictly adopted a case–control design—i.e. nest-tree samples were paired with nearby non-nest tree samples—we included the identity of each case–control pair as a random intercept. In our case, including this random intercept aimed to mimic a repeated measure analysis. We also included the circumference at breast height (CBH) of each nest and non-nest tree as a covariate to account for uncontrolled trait differences between the nest-tree and non-nest tree, even though all paired trees were, by definition, conspecific emergents (i.e. belonging to the same tree species). Models were run using the nlme package^58^ available in R 4.0.3, assuming a Gaussian residual distribution. Each model’s residuals were visually checked for normality and homoscedasticity.

## Supplementary Information

Supplementary Information.

## Data Availability

The data is available through the repository Mendeley Data (https://data.mendeley.com/9hgvn9s4nx).
