# Supplementary material for: Long-term concentration of tropical forest nutrient hotspots is generated by a central-place apex predator
Source: Sci Rep. 2023 Mar 17;13:4464. doi: 10.1038/s41598-023-31258-8 (PMC10023775; doi:10.1038/s41598-023-31258-8)

## Supplementary Information

**Supplementary information Figure S1.** Nest sites used to calculate nest density using both the polygon method and the maximum packed nest density method (MNPD). Nest densities were estimated at 1.55-3.30 nests/100km² of forest in panel A, and 1.97-4.84 nests/100km² of forest in panel B. Mapping data are based on 2019 Landsat imagery geoprocessed as part of the MapBiomas project (mapbiomas.org collection 5; year 2019; QGIS V.3.16; https://www.qgis.org).

**
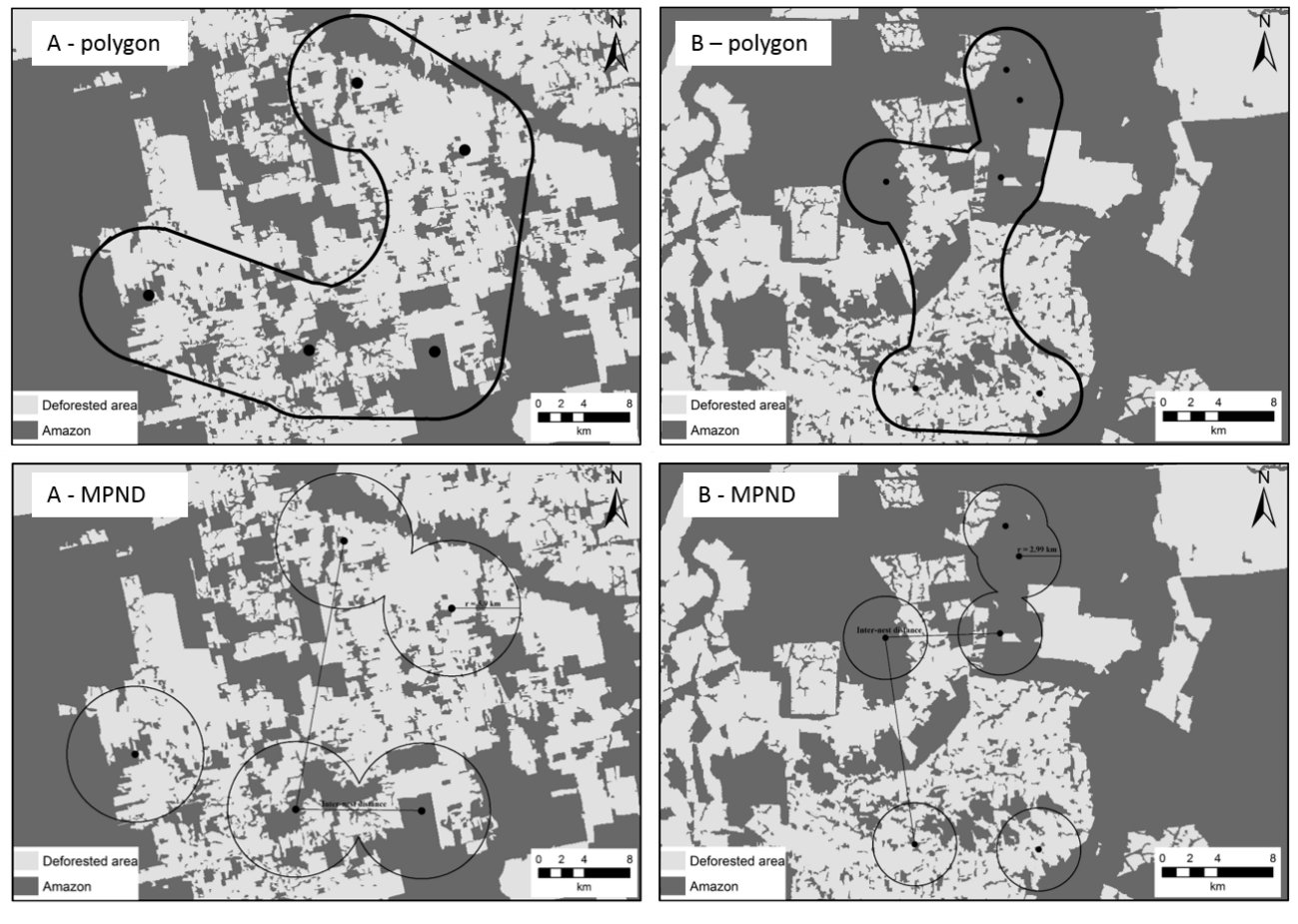
**

**Supplementary information Table S1.** Nest density calculate by maximum packed nest density (MPND) and polygon method, for two nest clusters in the study site.

|  | **Site** | **Nests** | **Total area (km²)** | **Forest area (km²)** | **Density (nests/100km²)** |
| --- | --- | --- | --- | --- | --- |
| MPND | |  |  |  |  |
|  | A | 5 | 632.85 | 253.66 | 1.97 |
|  | B | 6 | 195.12 | 123.9 | 4.84 |
| Polygon | | |  |  |  |
|  | A | 5 | 742.3 | 322.63 | 1.55 |
|  | B | 6 | 271.62 | 181.63 | 3.30 |

**Supplementary information Table S2.** Summary statistics of models ran for different nutrients in different stratum. Parameters of the model (parameter column), coefficient estimation (coefficient), lower and upper 95% interval confidence (lower and upper), degrees of freedom (df), t statistic (t-value) and probability associate (p-value).

| ***stratum*** | **nutrient** | **parameter** | **coefficient** | **lower** | **upper** | **df** | **t-value** | **p-value** |
| --- | --- | --- | --- | --- | --- | --- | --- | --- |
| *Soil* | Phosphorous | Intercept | 3.18 | 1.796 | 4.563 | 347 | 4.52 | <0.001 |
|  |  | Nest | -0.928 | -1.503 | -0.353 | 347 | -3.175 | 0.002 |
|  |  | Inactive | -0.594 | -1.532 | 0.344 | 347 | -1.246 | 0.214 |
|  |  | Circumference | 0.093 | -0.164 | 0.35 | 347 | 0.71 | 0.478 |
|  |  | Nest:inactive | 0.189 | -0.65 | 1.028 | 347 | 0.444 | 0.657 |
|  | Potassium | Intercept | 40.22 | 24.137 | 56.304 | 347 | 4.918 | <0.001 |
|  |  | Nest | -2.586 | -10.406 | 5.234 | 347 | -0.65 | 0.516 |
|  |  | Inactive | -7.916 | -17.931 | 2.1 | 347 | -1.555 | 0.121 |
|  |  | Circumference | 3.638 | 0.400 | 6.875 | 347 | 2.21 | 0.028 |
|  |  | Nest:inactive | 2.456 | -8.929 | 13.84 | 347 | 0.424 | 0.672 |
|  | Calcium | Intercept | 40.22 | 24.137 | 56.304 | 347 | 4.918 | <0.001 |
|  |  | Nest | -2.586 | -10.406 | 5.234 | 347 | -0.65 | 0.516 |
|  |  | Inactive | -7.916 | -17.931 | 2.1 | 347 | -1.555 | 0.121 |
|  |  | Circumference | 3.638 | 0.400 | 6.875 | 347 | 2.21 | 0.028 |
|  |  | Nest:inactive | 2.456 | -8.929 | 13.84 | 347 | 0.424 | 0.672 |
|  | Magnesium | Intercept | 40.22 | 24.137 | 56.304 | 347 | 4.918 | <0.001 |
|  |  | Nest | -2.586 | -10.406 | 5.234 | 347 | -0.65 | 0.516 |
|  |  | Inactive | -7.916 | -17.931 | 2.1 | 347 | -1.555 | 0.121 |
|  |  | Circumference | 3.638 | 0.400 | 6.875 | 347 | 2.21 | 0.028 |
|  |  | Nest:inactive | 2.456 | -8.929 | 13.84 | 347 | 0.424 | 0.672 |
|  | Alumnium | Intercept | 40.22 | 24.137 | 56.304 | 347 | 4.918 | <0.001 |
|  |  | Nest | -2.586 | -10.406 | 5.234 | 347 | -0.65 | 0.516 |
|  |  | Inactive | -7.916 | -17.931 | 2.1 | 347 | -1.555 | 0.121 |
|  |  | Circumference | 3.638 | 0.4 | 6.875 | 347 | 2.21 | 0.028 |
|  |  | Nest:inactive | 2.456 | -8.929 | 13.84 | 347 | 0.424 | 0.672 |
| *Undergrowth* | Nitrogen | Intercept | 19.911 | 13.662 | 26.16 | 48 | 6.407 | <0.001 |
|  |  | Nest | 2.033 | -1.415 | 5.481 | 48 | 1.186 | 0.242 |
|  |  | Inactive | -2.643 | -5.39 | 0.104 | 18 | -2.021 | 0.058 |
|  |  | Circumference | 0.19 | -1.233 | 1.612 | 48 | 0.268 | 0.79 |
|  |  | Nest:inactive | 2.489 | -2.569 | 7.546 | 48 | 0.989 | 0.327 |
|  | Phosphorous | Intercept | 0.173 | 0.038 | 0.308 | 48 | 2.569 | 0.013 |
|  |  | Nest | 0.037 | -0.02 | 0.094 | 48 | 1.31 | 0.196 |
|  |  | Inactive | 0.034 | -0.025 | 0.092 | 18 | 1.212 | 0.241 |
|  |  | Circumference | -0.006 | -0.036 | 0.025 | 48 | -0.374 | 0.71 |
|  |  | Nest:inactive | 0.015 | -0.069 | 0.098 | 48 | 0.355 | 0.724 |
|  | Potassium | Intercept | 13.031 | 7.871 | 18.19 | 48 | 5.078 | <0.001 |
|  |  | Nest | 2.151 | 0.209 | 4.093 | 48 | 2.227 | 0.031 |
|  |  | Inactive | 0.533 | -1.733 | 2.8 | 18 | 0.494 | 0.627 |
|  |  | Circumference | -0.007 | -1.162 | 1.148 | 48 | -0.012 | 0.99 |
|  |  | Nest:inactive | -3.172 | -6.027 | -0.318 | 48 | -2.234 | 0.03 |
| *Nest-tree* | Nitrogen | Intercept | 19.911 | 13.662 | 26.16 | 48 | 6.407 | <0.001 |
|  |  | Nest | 2.033 | -1.415 | 5.481 | 48 | 1.186 | 0.242 |
|  |  | Inactive | -2.643 | -5.39 | 0.104 | 18 | -2.021 | 0.058 |
|  |  | Circumference | 0.19 | -1.233 | 1.612 | 48 | 0.268 | 0.79 |
|  |  | Nest:inactive | 2.489 | -2.569 | 7.546 | 48 | 0.989 | 0.327 |
|  | Phosphorous | Intercept | 0.173 | 0.038 | 0.308 | 48 | 2.569 | 0.013 |
|  |  | Nest | 0.037 | -0.02 | 0.094 | 48 | 1.31 | 0.196 |
|  |  | Inactive | 0.034 | -0.025 | 0.092 | 18 | 1.212 | 0.241 |
|  |  | Circumference | -0.006 | -0.036 | 0.025 | 48 | -0.374 | 0.71 |
|  |  | Nest:inactive | 0.015 | -0.069 | 0.098 | 48 | 0.355 | 0.724 |
|  | Potassium | Intercept | 13.031 | 7.871 | 18.19 | 48 | 5.078 | <0.001 |
|  |  | Nest | 2.151 | 0.209 | 4.093 | 48 | 2.227 | 0.031 |
|  |  | Inactive | 0.533 | -1.733 | 2.8 | 18 | 0.494 | 0.627 |
|  |  | Circumference | -0.007 | -1.162 | 1.148 | 48 | -0.012 | 0.99 |
|  |  | Nest:inactive | -3.172 | -6.027 | -0.318 | 48 | -2.234 | 0.03 |
| *Canopy surrounding* | Nitrogen | Intercept | 19.365 | 15.062 | 23.668 | 194 | 8.877 | <0.001 |
|  |  | Nest | 16.95 | 14.385 | 19.515 | 194 | 13.033 | <0.001 |
|  |  | Inactive | 0.79 | -1.732 | 3.313 | 18 | 0.658 | 0.519 |
|  |  | Circumference | -0.213 | -1.159 | 0.733 | 194 | -0.444 | 0.657 |
|  |  | Nest:inactive | 4.197 | 0.335 | 8.059 | 194 | 2.143 | 0.033 |
|  | Phosphorous | Intercept | 0.225 | 0.127 | 0.322 | 194 | 4.561 | <0.001 |
|  |  | Nest | 0.319 | 0.268 | 0.371 | 194 | 12.241 | <0.001 |
|  |  | Inactive | 0.023 | -0.039 | 0.086 | 18 | 0.787 | 0.442 |
|  |  | Circumference | -0.008 | -0.029 | 0.013 | 194 | -0.78 | 0.437 |
|  |  | Nest:inactive | -0.012 | -0.09 | 0.065 | 194 | -0.312 | 0.755 |
|  | Potassium | Intercept | 12.808 | 9.209 | 16.408 | 194 | 7.018 | <0.001 |
|  |  | Nest | 10.116 | 7.717 | 12.515 | 194 | 8.316 | <0.001 |
|  |  | Inactive | 0.303 | -1.715 | 2.322 | 18 | 0.316 | 0.756 |
|  |  | Circumference | 0.213 | -0.583 | 1.01 | 194 | 0.528 | 0.598 |
|  |  | Nest:inactive | -7.498 | -11.115 | -3.881 | 194 | -4.089 | <0.001 |

**Supplementary information Figure S2.** Map showing the geographic location of all 20 harpy eagle nests (white circles) sampled in terms of soil and plant chemistry within our southern Amazonian study region in northern Mato Grosso. Prey composition and delivery rates was also monitored at all active nests for up to 24 months. Background map shows forest cover (dark gray) and deforestation areas (light gray). Mapping data are based on 2019 Landsat imagery geoprocessed as part of the MapBiomas project (mapbiomas.org collection 5; year 2019; QGIS V.3.16; https://www.qgis.org).


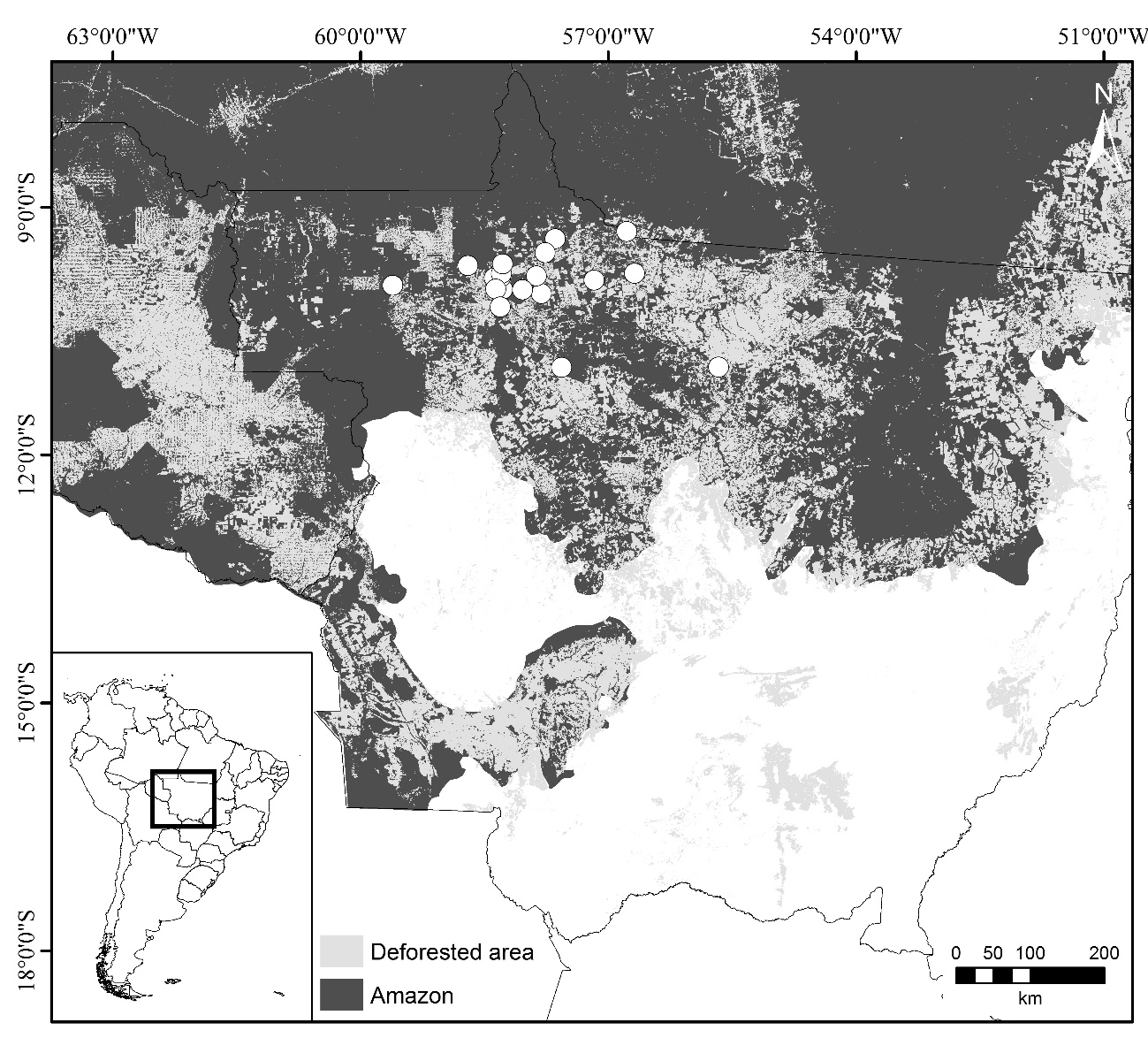

Supplement: Supplementary file 1 — Supplementary Information. [file 41598_2023_31258_MOESM1_ESM.docx]
